# Nature and Treatment of Comorbid Alcohol Problems and Post-Traumatic Stress Disorder Among American Military Personnel and Veterans

**DOI:** 10.35946/arcr.v38.1.16

**Published:** 2016

**Authors:** John P. Allen, Eric F. Crawford, Harold Kudler

**Affiliations:** John P. Allen, Ph.D., M.P.A., is a consultant, Eric F. Crawford, Ph.D., is the assistant director of the Clinical Core, and Harold Kudler, M.D., is an associate director; all at the VA Mid-Atlantic Health Care Network Mental Illness Research, Education and Clinical Center Durham, North Carolina. Dr. Allen also is a consulting professor, Dr. Crawford is an assistant professor, and Dr. Kudler is an associate professor in the Department of Psychiatry and Behavioral Sciences at Duke University Medical Center, Durham, North Carolina

**Keywords:** Alcohol use, abuse and dependence, problematic alcohol use, post-traumatic stress disorder, stress, military, veterans, combat exposure, treatment

## Abstract

Many service members and veterans seeking treatment for alcohol problems also have post-traumatic stress disorder (PTSD). This article considers the effectiveness of treating alcohol problems and PTSD simultaneously. The authors begin by summarizing the extent of excessive alcohol use among military service members and veterans. They then explore the relationship between combat exposure and subsequent alcohol use; identify and briefly describe evidence-based treatments for alcohol problems and PTSD, separately; and review research on the effects of single treatments for both PTSD symptoms and alcohol use.

Many service members and veterans seeking treatment for alcohol problems have experienced the life-threatening stress of combat, many have post-traumatic stress disorder (PTSD), and many service members and veterans seeking treatment for PTSD have alcohol or other substance problems. Sensitivity to these issues can influence how a therapist relates to the patient and also has possible implications for developing a treatment strategy (U.S. Department of Veterans Affairs [DVA] 2010). Historically, clinicians have been concerned that patients need to reduce or resolve substance abuse before PTSD treatment can be successful. But research is showing that both disorders can be treated simultaneously. Here, we assess the scope of the problem and examine treatments that can be effective for treating each disorder individually as well as in tandem.

## Alcohol Problems in Active-Duty Military Personnel and Veterans

For more than 30 years the Department of Defense (DoD) has conducted recurrent surveys to determine rates of excessive alcohol use among active-duty personnel. The most recent of these (DoD 2013) revealed wide prevalence of “binge” drinking, defined as consuming 5 or more drinks for males or 4 or more drinks for females on a single occasion. An analysis of this survey by Bray and colleagues (2013) found that across the U.S. Armed Services 33 percent of personnel reported binge drinking during the 30 days preceding the survey, with considerable variation in rates across military departments (Army, 34 percent; Navy, 38 percent; Marines, 49 percent; and Air Force, 24 percent). Twenty percent of male and female active-duty personnel engaged in heavy drinking, which was defined as binge drinking at least once a week during the past 30 days (Bray et al. 2013).

Less is known about alcohol use problems among veterans. One analysis examined results from the National Survey on Drug Use and Health from 2004 through 2010 (Golub et al. 2013). The study compared veterans ages 21 to 34 with non-veteran peers matched on age and gender. The two groups were quite similar in their rates of alcohol use disorder (AUD) in the past year (15 percent); “binge” drinking (44 percent), defined as consuming 5 or more drinks on at least one occasion during the past 30 days; and heavy drinking (14 percent), defined as binge drinking on 5 or more days during the past 30 days (Golub et al. 2013).

## Combat Stress and Alcohol Misuse

As of September 30, 2013, 2.6 million service members had been deployed to Operation Enduring Freedom, Operation Iraqi Freedom, and Operation New Dawn since 2001 (DVA 2013). Due to high rates of combat and blast exposure, healthcare providers within the DOD and the U.S. Departments of Veterans Affairs (VA) are offering services to increasing numbers of veterans and active-duty personnel returning with complex mental and physical health problems (Hoge et al. 2004, 2008).

PTSD is the most common mental health diagnosis for the nearly 1 million U.S. veterans who served in Iraq and Afghanistan between October 1, 2001, and September 30, 2013, and who accessed services through the Veterans Health Administration (VHA) (DVA 2013). Nineteen percent of those who have served in Iraq and Afghanistan develop PTSD within a year of their return to the United States (Tanielian and Jaycox 2008).

Symptom clusters for PTSD as defined by the *Diagnostic and Statistical Manual of Mental Disorders* (DSM–5) are illustrated in the accompanying textbox (American Psychiatric Association 2013). Based on the previous DSM–IV criteria (American Psychiatric Association 1994), rates of PTSD in returning service members vary somewhat as a function of the method for collecting data, with results from screening instruments suggesting a range of 10 to 20 percent (Milliken et al. 2007; Seal et al. 2007; Sundin et al. 2010). Structured clinical interviews yield a somewhat lower but still disconcerting PTSD rate of 7 to 10 percent (Erbes et al. 2007). Among individuals with a history of traumatic brain injury, rates of PTSD seem to escalate to 33 to 39 percent (Carlson et al. 2011). An analysis of VA healthcare statistics from October 7, 2001, to March 31, 2008, showed that PTSD was the most prevalent psychiatric diagnosis, affecting approximately 21.5 percent of patients (Cohen et al. 2010). As of 2014, VA public health data suggest that 30 percent of veterans of military service in Afghanistan and Iraq seeking VA care have PTSD.

**DSM–5 Post-Traumatic Stress Disorder Symptom Clusters**

***Re-experiencing***

- Recurrent, intrusive, and distressing memories, images, thoughts, and/or perceptions
- Recurrent distressing dreams
- Dissociative reactions (flashbacks)
- Marked psychological and/or physiological response to cues that symbolize or resemble the event

***Avoidance***

- Of memories, thoughts, or feelings about the event
- Of reminders of the event

***Negative Alterations in Cognitions and Mood***

- Inability to recall an important aspect of the event
- Persistent, exaggerated negative beliefs or expectations about self, others, or the world
- Persistent negative emotional state
- Diminished interest/participation in significant activities
- Detachment/estrangement
- Persistent inability to experience positive emotions

***Marked Alterations in Arousal and Reactivity***

- Irritability/outbursts or anger
- Reckless or self-destructive behavior
- Hypervigilance
- Exaggerated startle response
- Difficulty concentrating
- Difficulty falling or staying asleep or restless sleep

Substance use disorders (SUDs) are another common reason for seeking mental health services. PTSD and substance use disorder frequently co-occur (McCauley et al. 2012). As illustrated by the figure, a consistently increasing percentage of veterans who have received VHA care, regardless of when they served in the military, have been diagnosed as having comorbid PTSD and SUD. In fiscal year 2013, 26.5 percent of VA patients with a diagnosis of PTSD also had SUDs. It is also worth noting that the number of veterans with both conditions has increased by 76 percent since fiscal year 2008, a rate exceeding the increase in prevalence for PTSD (52.3 percent) or for SUD (33.1 percent) alone (Program Evaluation and Resource Center, VA Medical Center, Palo Alto, CA. January 2014, personal correspondence).

Individuals with AUD and PTSD tend to have greater risks for other psychiatric disorders, respond less favorably to interventions for the AUD, and are at increased risk of relapse to problematic drinking (Torchalla et al. 2012).

## Relationship between PTSD and Substance Misuse

Citing data from the National Comorbidity Survey (Kessler et al. 1995), Jacobsen and colleagues (2001) observed that, when they exclude nicotine dependence, the psychiatric condition most likely to co-occur among men with PTSD was alcohol abuse/dependence. Among women with PTSD, alcohol abuse/dependence was the second most common mental health combination, with depression or anxiety being the most common. Study investigators proposed two reasons for this association. For one, PTSD may follow alcohol misuse, because people who misuse alcohol may tend to place themselves in situations that involve increased risk for trauma and subsequent PTSD; alcohol may also sensitize them to developing a PTSD reaction in response to trauma. Second, alcohol misuse may follow PTSD by playing a “self-medication” role to dampen the hyperarousal component of PTSD. Interestingly, Jacobsen and colleagues further comment that the neuronal arousal associated with alcohol withdrawal may be augmented by PTSD-linked hyperarousal and may make individuals with PTSD more likely to return to drinking than those who need only cope with the arousal associated with acute drinking cessation.

A study of patients receiving treatment for SUD indicated that improvements in PTSD symptoms over 2-week periods during the 26-week study were associated with decreases in cocaine and opioid use and possibly reductions in alcohol use (p=.056) (Ouimette et al. 2010). These findings support the theory that people with PTSD use drugs and alcohol to self-medicate. However, the study sample was small and consisted solely of patients currently in treatment. Hence, the finding may not generalize well to a random sample of people with both conditions.

## Combat and Subsequent Alcohol Misuse

Milliken and colleagues (2007) conducted the largest study of combat’s influence on mental health functioning of service members. They analyzed responses on the Post Deployment Health Reassessment (PDHRA), a clinical and self-report measure that includes questions related to combat stress and alcohol problems. Soldiers completed the survey 3 to 6 months after redeployment to combat service in Iraq. More than 88,000 soldiers completed both this survey and a related-content survey administered to them at redeployment. Nearly 70 percent of respondents reported traumatic combat experiences, and around 50 percent of active personnel and reserve component personnel reported that at some time they feared that they would be killed. Nine percent of active-duty respondents and 14 percent of U.S. Army Reserve and National Guard soldiers endorsed at least three of four PTSD screening items. The PDHRA also included a two-item screen for alcohol problems; 12 percent and 15 percent, respectively, of the active duty and reserve component respondents endorsed at least one such item. Yet only 0.4 percent of the sample reported having been referred to substance abuse treatment.

Data from the large-scale Air Force Community Assessment Survey conducted in the spring of 2008 demonstrated a relationship between the total number of deployments and cumulative time deployed with the subsequent likelihood of an Air Force member becoming a problem drinker. Each additional year of deployment increased the risk of becoming a problem drinker by 23 percent, and each additional deployment period increased the risk by 14 percent. Interestingly, the risk of becoming a problem drinker was not associated with how recently a soldier was deployed (Spera et al. 2011).

Another survey (Santiago et al. 2010) given to soldiers 3 to 4 months after returning from deployment to Iraq found that 27 percent scored positive for alcohol misuse, as shown by endorsement of at least one of two screening items on the Two-Item Conjoint Screen. Soldiers exposed to more intense combat were also more likely to score positive on the alcohol misuse screen. Another study found that deployments involving combat exposure also were associated with post-deployment heavy weekly drinking, binge drinking, and alcohol-related problems among active duty and reserve component personnel (Jacobson et al. 2008).

Alcohol problems among military personnel exceed those of civilian populations in part because of demo-graphic differences in age, gender balance, and education level among military populations. However, other factors contribute to the risk of alcohol misuse among service members, including deployment stress, combat exposure, and PTSD. Reflecting this, an increasing number of veterans are being treated by the VHA for comorbid SUDs and PTSD. The challenge is to implement treatments found to be effective for both conditions, as well as to continue to develop more effective interventions.

## Effective Alcohol Treatments

### Psychotherapies

Several psychosocial interventions for treating alcohol problems have shown strong evidence for effectiveness. The VHA’s policy is that patients with alcohol problems have access to at least two of the following:

- *Cognitive–Behavioral Therapy for Relapse Prevention,* which assists patients in identifying internal and external stimuli that prompt drinking, and in learning skills and alternative ways of thinking to cope with these cues and avoid alcohol use.
- *12-Step Facilitation,* which promotes participation in Alcoholics Anonymous and working the steps of the program. It employs a treatment manual with activities and homework assignments and is conducted in a one-on-one counseling relationship.
- *Community Reinforcement Approach,* which helps patients establish a strong environmental support system to help sustain sobriety.
- *Substance Use Disorder–Focused Behavioral Couples Counseling/Family Therapy,* which emphasizes the participation of significant others in treatment. Sessions focus on improvements in communication and interactional patterns of the couple or family, especially as they relate to drinking.
- *Motivational Enhancement Therapy,* which builds on principles of motivational interviewing. It employs treatment processes that reflect the patient’s level of readiness for change.

For detailed descriptions of these treatments, see Finney and Moos (2002).

## Pharmacotherapies

The *VA/DoD Clinical Practice Guideline for Management of Substance Abuse Disorders* (DVA and DoD 2010) offers the following recommendations for the pharmacological management of alcohol dependence:

- Oral naltrexone should be routinely considered in conjunction with addiction counseling.
- Injectable naltrexone is effective in conjunction with addiction counseling when the patient is willing to accept monthly injections.
- Acamprosate should routinely be considered in conjunction with addiction counseling as an alternative to naltrexone.
- Disulfiram should only be used when the goal is abstinence.

A recent meta-analysis reinforces the value of pharmacological treatment for alcohol abuse (Jonas et al. 2014). The analysis found that both acamprosate and oral naltrexone were associated with reductions in how often patients returned to drinking with no significant differences between the two drugs in controlling alcohol consumption. The authors emphasize that less than one-third of people with AUD receive treatment, and only a small percentage of these patients (less than 10 percent) receive medications to assist in reducing alcohol consumption. A companion editorial by Bradley and Kivlahan (2014) emphasizes the importance of integrating psychopharmacological and psychosocial interventions in treating AUD and of integrating these treatments into primary care services.

## Effective PTSD Treatments

### Psychotherapies

In 2008, the Institute of Medicine conducted a comprehensive review of outcomes on existing PTSD treatments. The report determined that “evidence is sufficient to conclude the efficacy of exposure therapies in the treatment of PTSD” (chapter 4, p. 97). Shortly thereafter, the VHA began promoting the use of two trauma-focused, manualized cognitive–behavioral psychotherapies (Karlin et al. 2010): Prolonged Exposure (PE; Foa et al. 2007) and Cognitive Processing Therapy (CPT; Resick and Schnicke 1992). Both interventions demonstrated efficacy in randomized controlled trials with civilians (Foa et al. 1999, 2005; Resick et al. 2002) and veterans (Monson et al. 2006; Schnurr et al. 2007). Evidence for both psychotherapies for veterans and active duty service members has continued to accumulate (Chard et al. 2010; Goodson et al. 2013; Rauch et al. 2009; Tuerk et al. 2011; Walter et al. 2014). Treatment effectiveness seems to persist following treatment (Resick et al. 2012). The goals of both interventions are to reduce avoidant coping; purposefully confront traumatic memories; and modify maladaptive, trauma-related thoughts. Nevertheless, the rationales and procedures of the two treatments differ significantly.

PE includes four essential elements: psychoeducation, in-vivo exposure, imaginal exposure, and in-session discussion following imaginal exposures to facilitate emotional processing and corrective learning (Foa et al. 2007). In the initial phase of treatment, therapists present information about common reactions to trauma, factors that maintain PTSD symptoms, conceptual bases for interventions, and breathing retraining. They reinforce this information with standardized handouts. In-vivo exposure procedures require patients to progressively confront situations and stimuli (including sights and sounds) that they previously avoided, because they associated the situations and stimuli with their traumatic memory. Imaginal exposure asks patients to verbally revisit their traumatic memory and emotionally process the experience to bring about corrective learning and habituation in later treatment sessions. Imaginal exposure begins in the third session and is followed by a collaborative “processing” discussion, typically involving support, normalization of experience, and discussion about key perceptions linked with the traumatic experience. In the mid-to-later phases of PE, imaginal exposure focuses on the most distressing aspects of the index trauma, or “hotspots.” Patients typically complete 90-minute sessions once a week, with most patients requiring 8 to 15 sessions for treatment completion. Clinicians audiotape sessions and require patients to review the tapes between appointments.

CPT (Resick 2001) consists of 12 treatment sessions that include cognitive interventions in either a group or individual format. During the initial sessions, patients receive psycho-education about PTSD and underlying information processing frameworks, complete written assignments to clarify the personal significance of traumatic experiences, and identify problematic trauma-related beliefs or “stuck points.” During the middle stages of CPT, patients learn to use a variety of worksheets to identify linkages between events, thoughts, and feelings; to produce and repeatedly read detailed accounts of their most traumatic experience(s), with an emphasis on experiences associated with traumatic events; and to begin challenging their stuck points with support and assistance from the therapist. Therapists use Socratic questioning to teach patients to examine and modify relevant maladaptive cognitions that maintain PTSD symptoms. They assign patients daily worksheets for home practice. In the final phases of the treatment, therapists aim to modify beliefs in five key domains: safety, trust, power/control, esteem, and intimacy. Patients consolidate their treatment gains in the concluding session.

### Pharmacotherapies for PTSD

A wide range of psychotropic medications have been explored for treating PTSD. *VA/DoD Clinical Practice Guidelines for the Management of Post-Traumatic Stress* (DVA and DoD 2010) most strongly recommend selective serotonin reuptake inhibitors (SSRIs) and serotonin norepinephrine reuptake inhibitors (SNRIs). The high blood pressure medication, prazosin, has been increasingly used to treat PTSD, but the *VA/DoD Guidelines* only recommend this as an adjunctive therapy for nightmares associated with the disorder.

## Treating Co-Occurring PTSD and AUD

### Psychosocial Treatments

Few well-controlled studies have assessed the efficacy of trauma-focused, cognitive–behavioral treatments, such as PE or CPT, in patients dually diagnosed with PTSD and SUD or AUD. This likely reflects a bias toward excluding patients with dual diagnosis from clinical trials because of traditional clinical concerns that concurrent misuse of substances could diminish the benefits of PTSD treatment (Riggs et al. 2003), or that exposure-based interventions might lead to relapse or to escalation of substance misuse (Hien et a. 2004; McGovern et al. 2009).

Taken in concert, the literature on treatments for co-occurring PTSD and AUD indicates that dually diagnosed patients can tolerate and benefit from psychotherapies specifically formulated to address trauma and PTSD. In fact, a forthcoming meta-analytic *Cochrane Review* that consolidates outcomes from over 1,400 participants (Roberts et al. 2012) concludes that combined, trauma-focused interventions meant to address both PTSD and AUD or SUD perform as well as or better than usual treatments in reducing symptoms of both disorders. Nonetheless, there is room for much improvement in this area, and debate continues about how best to engage and treat this complex population (Foa et al. 2013*b*; Najavits 2013). Additional research also is needed to determine optimal methods for assisting veterans or service members with co-occurring conditions and retaining them in treatment.

Several descriptions and reports also have been published on the use of present-focused, skills-based psychotherapies specifically targeted to the needs of dually diagnosed patients. Of these, Seeking Safety, a manualized cognitive–behavioral treatment that can be delivered to individuals or groups, has received the greatest attention (Najavits and Hein 2013; Najavits et al. 1998). Each session includes components for reducing the effects of trauma (“safety”) and diminishing substance use and follows the same structure: a “check-in” where therapists gather information on maladaptive or “unsafe” behaviors and coping skills among patients; a review of a quotation that captures the essence of the current session’s topic; a review of handouts to facilitate discussion and skills practice linked with the topic; and a “check-out” asking patients to commit to between-session skills implementation. The full protocol includes sessions dealing with 25 different topics, including promoting safety, taking back power from PTSD, healing from anger, creating meaning, and detaching from emotional pain or grounding. The protocol does not include any exposure-based exercises.

Although participants have generally accepted Seeking Safety and 22 reports have found mostly beneficial outcomes with PTSD-related symptoms and alcohol or substance use (Najavits and Hien 2013), the largest controlled trial evaluating this treatment found null results when contrasted with a health education control protocol. There is also a high rate of attrition among patients receiving Seeking Safety (Hien et al. 2009). The few studies of Seeking Safety conducted with veterans have included small sample sizes of not more than 25 patients each (Cook et al. 2006; Norman et al. 2010). Seeking Safety also has often failed to outperform control conditions on outcome measures for PTSD (Boden et al. 2012) or substance use (Desai et al. 2008). It thus remains uncertain whether this treatment should be considered a treatment of choice for veterans or military service members with co-occurring PTSD and AUD. However, for those who do not choose to begin trauma-focused therapy, Seeking Safety can be an effective engagement strategy that may be sufficient to reduce symptoms for some and to act as an effective preparation for trauma-focused treatment for others.

### Psychopharmacologic Treatments

Less is known about the clinical value of combining pharmacological treatments with psychosocial treatments for co-occurring PTSD and alcohol dependence (Ravelski et al. 2014), but an article from Foa and colleagues (2013*a*) suggests that combining prolonged exposure therapy and oral naltrexone may be effective in reducing the percentage of drinking days in this population.

There are no direct contraindications to prescribing patients with PTSD any of the pharmacotherapeutic agents recommended in the *VA/DoD Clinical Practice Guidelines for the Management of Substance Use Disorders* (DVA and DoD 2009) for the treatment of AUD. However, certain other conditions commonly associated with PTSD and alcohol dependence may preclude use of some pharmaceuticals. For example, if patients have sustained significant liver damage subsequent to co-existing PTSD and alcohol dependence, they should avoid naltrexone and disulfiram. In addition, intravenous substance abuse may contribute to renal disease, which may complicate the use of naltrexone or acamprosate. Findings that PTSD itself may predispose patients to coronary artery disease (Edmondson et al. 2013) suggest that a careful cardiac evaluation be performed before prescribing disulfiram. Finally, chronic pain frequently co-occurs with both PTSD and substance abuse, and naltrexone may interfere with currently effective pain control regimens that rely on opioid agents.

Benzodiazepines are an effective treatment for relieving symptoms of alcohol withdrawal. However, the VA/DoD PTSD guidelines (DVA and DoD 2010) raise concerns about using benzodiazepines to treat PTSD, because these agents have not been shown to be effective as single-channel treatments for PTSD and might even complicate PTSD’s course. Although this is not an absolute contraindication to the acute use of benzodiazepines for alcohol detoxification, it does call for careful monitoring of any ongoing benzodiazepine use. Along these same lines, clinicians should consider the severe physiological stress that can be associated with future states of intoxication and withdrawal when they choose a treatment for patients with combined PTSD and alcohol dependence who are prone to withdrawal. For example, use of a tricyclic anti-depressant to treat PTSD (not a top recommendation in the VA/DoD PTSD guidelines (DVA and DoD 2010), but a treatment that can be effective for PTSD) may lower seizure threshold in a patient prone to cycles of alcohol relapse and withdrawal. Also, prazosin, which was originally marketed as an antihypertensive, could cause hypotension in medically unstable patients, including during states of dehydration or in patients in alcohol withdrawal.

Although the 2010 VA/DoD Clinical Practice Guideline for the Management of Post-Traumatic Stress lists topiramate as having no demonstrated benefit for PTSD, a pilot study suggests that this anticonvulsant may have some value for treating both PTSD and AUD (Batki et al. 2014). However, topiramate cannot be recommended currently as a first- or second-line treatment for either disorder.

## Conclusion

AUD and PTSD are common and severe problems in veterans and military service members and merit intervention. Fortunately, a number of psychological treatments and medications have been demonstrated as effective for each problem and should be incorporated into clinical practice whether the conditions occur independently or together. When AUD and PTSD occur in the same patient, they should generally be addressed simultaneously, either in closely coordinated or integrated care. Contrary to earlier clinical concerns that substance abuse should be reduced or resolved before treatment for PTSD, it seems that for most patients the treatments can be performed simultaneously with good results. In fact, clinical experience and emerging research suggests that it is best to combine modalities and targets within a comprehensive treatment plan. As in other areas of clinical practice, clinicians should systematically and frequently monitor patient progress to determine if some modification may be needed in the treatment protocol. It also is important to assess the patient’s medical status before prescribing pharmacotherapies. In many cases, especially those involving alcohol dependence, adjunct medications will prove useful.

## Figures and Tables

**Figure:**
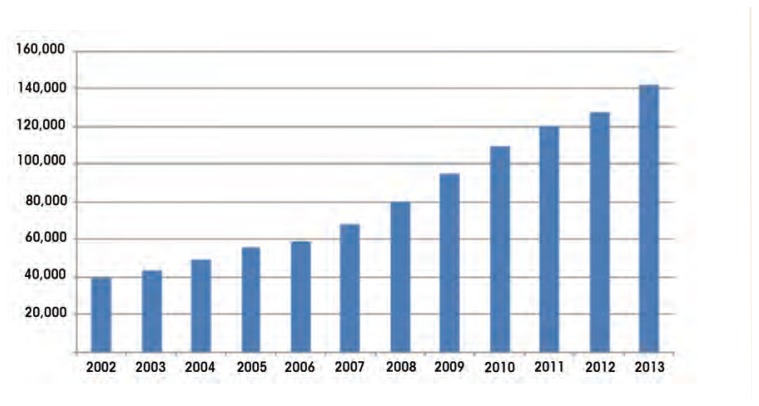
Veterans receiving care in the Veterans Health Care Administration for comorbid PTSD and substance use disorder by year. SOURCE: Program Evaluation and Resource Center, VA Medical Center, Palo Alto, CA. January 2014, personal correspondence.
